# Behavioral Effects of Upper Respiratory Tract Illnesses: A Consideration of Possible Underlying Cognitive Mechanisms

**DOI:** 10.3390/bs2010038

**Published:** 2012-03-15

**Authors:** Andrew P. Smith

**Affiliations:** School of Psychology, Cardiff University, Centre for Occupational and Health Psychology, 63 Park Place, Cardiff CF10 3AS, UK; E-Mail: SmithAP@Cardiff.ac.uk; Tel: +44-29-20874757; Fax: +44-29-20874758

**Keywords:** upper respiratory tract illness, common cold, reaction time, alertness, working memory

## Abstract

Previous research has shown that both experimentally induced upper respiratory tract illnesses (URTIs) and naturally occurring URTIs influence mood and performance. The present study investigated possible cognitive mechanisms underlying the URTI-performance changes. Those who developed a cold (N = 47) had significantly faster, but less accurate, performance than those who remained healthy (N = 54). Illness had no effect on manipulations designed to influence encoding, response organisation (stimulus-response compatilibility) or response preparation. Similarly, there was no evidence that different components of working memory were impaired. Overall, the present research confirms that URTIs can have an effect on performance efficiency. Further research is required to identify the physiological and behavioral mechanisms underlying these effects.

## 1. Introduction

Minor illnesses, such as influenza and the common cold are frequent, widespread and a major cause of absenteeism from work and education. Research also suggests that these illnesses impair mental efficiency and influence mood. The effects of influenza and the common cold are rather different and one cannot generalize from one type of infection to all upper respiratory tract illnesses. Initial evidence for effects of upper respiratory tract illnesses on performance came largely from case histories and anecdotal reports [1,2]. Investigations of effects of experimentally induced influenza and colds have confirmed that these illnesses influence performance and mood [3,4]. Influenza impaired detection of stimuli presented at uncertain times and in unknown locations [5,6]. These effects can be mimicked by giving injections of alpha interferon [7], a cytokine known to impair frontal lobe functioning [8,9]. An effect of influenza on frontal lobe functioning is supported by studies showing impairments on executive tasks, such as the Stroop task [10]. The effects of influenza have been replicated in a study of naturally occurring illnesses that used virological techniques to assess the nature of the infecting agent [11]. Other upper respiratory tract illnesses that lead to systemic symptoms (e.g., fever, myalgia) have been shown to produce very similar effects to those of influenza (e.g., infectious mononucleosis [12]).

The effects of colds on performance appear to be different from those of influenza. Studies of experimentally induced colds have shown that volunteers with colds have impaired psychomotor functioning (poor hand-eye co-ordination, slower response times in reaction time tasks [5,13,14]). Again, these findings have been replicated in studies of naturally occurring colds [15,16,17,18,19,20,21,22,23]. Some studies have used virological assays [20] to ensure that upper respiratory tract infections are being studied. The effects have also been replicated in different laboratories [23] and, in general, the results from studies of naturally occurring colds replicate those seen with experimentally induced colds. There are two exceptions where effects of naturally occurring colds appear to be different. Simple reaction time and sustained attention have been found to be impaired in studies of naturally occurring colds. These effects were not observed with experimentally induced illnesses but this probably reflects the limited data collected using these tasks. 

The profile of the colds induced behavioral changes has similarities to those observed in other low arousal states (e.g., fatigue [24]). Indeed, an investigation of the effects of colds on speed of eye movements [19] has shown a similar slowing to that observed with sedative drugs. The reduction in arousal seen in those with a cold could be produced in several ways. First, it may reflect reduced stimulation from the sensory afferent nerves [25,26]. Secondly, it could reflect neurotransmitter changes produced by immunological responses to the viral infection. Indeed, research suggests that compounds that increase central noradrenaline (e.g., caffeine [18], idazoxan [21]) remove the drowsiness and psychomotor slowing associated with having a cold. People with upper respiratory illnesses have also been found to be more sensitive to other factors that can change alertness (e.g., noise [15]; alcohol [16]; fatigue [22]). Overall, these lines of evidence suggest that having a cold leads to a reduction in alertness due to changes in neurotransmitters that influence arousal. Infection may influence neurotransmitters in several ways and further research is required to investigate whether neuroendocrine or cytokine changes are responsible for the reduced arousal state. 

Other possible mechanisms have been suggested. Drake *et al.* [27] investigated whether impaired sleep underlies the performance changes seen in those with colds. Sleep disturbance has been found in volunteers with colds [27,28] but the effects are modest and it is unlikely that they can account for the performance effects. Indeed, sleep disturbance probably reflects symptoms such as cough or nasal congestion. The performance impairments associated with a cold appear to be largely independent of symptom severity [29] but it is unclear whether different types of symptoms have selective effects. Similarly, the performance impairments do not reflect mood changes [29].

Once the profile of impairments associated with having a cold has been established it is important to determine which mechanisms underlie these effects of illness on cognition. Little is known of the cognitive processes that are impaired and yet there are established models that can be used to address this issue. The following examples show how one can examine which cognitive processes are impaired in working memory and choice reaction time tasks. This approach has been adopted to examine mechanisms underlying other changes in state (e.g., effects of caffeine and breakfast [30]). Baddeley’s working memory model has been described in detail in several publications [31]. The model involves fractionating working memory into at least three sub-components: the visuo-spatial scratchpad, the articulatory loop and the central executive. The visuo-spatial scratchpad is assumed to manipulate visuo-spatial information and is regarded as responsible for visual imagery. The articulatory loop performs a similar function for speech-based information. These systems are co-ordinated and linked to long-term memory by an attentional control system, the central executive. It has been relatively easy to develop tests to examine the articulatory loop and visuo-spatial scratchpad. For example, serial recall of the order of digits (and reverse recall) and recall of words of different lengths provide measures of the efficiency and rate of articulation. Similarly, it is possible to study the visuo-spatial scratchpad using block tapping tasks and the pattern span technique. It has proved more difficult to assess the attentional controller, the central executive. However, a running memory task, where the subject has to recall in order the last eight items of a series but does not know when the series will stop does measure the efficiency of this part of working memory. 

Sanders [32] has argued that by systematically changing task variables in choice reaction time tasks one can determine whether effects are due to changes at the encoding, central or output stages of information processing. For example, by degrading the quality of visual stimuli, input related encoding is manipulated. If the effects of illness on choice reaction time are at this stage then an interaction between illness and stimulus degradation conditions should be observed. Similarly, central processing or response selection can be examined by varying the compatibility between stimulus and response and the output side of the information processing system can be investigated by varying the time uncertainty (the inter-stimulus interval). Interactions between illness and these task parameters would indicate that the illness influences these stages of processing. 

The present experiment used these two approaches to provide further information on mechanisms underlying effects of upper respiratory tract illnesses on performance. In this type of research it is usual to study the common cold rather than influenza. The infecting agent was not known in the present study and the nature of the illness was inferred from the symptoms.

## 2. Method

This study was carried out with the approval of the ethics committee, School of Psychology, Cardiff University.

### 2.1. Participants

A total of 101 participants, 35 males and 66 females, took part in this study. The age range of the sample was 18–27 years, with a mean age of 20.36 years. 

### 2.2. Exclusion Criteria

Several exclusion criteria were applied for recruitment of participants in this investigation. Participants were excluded if they were taking medication (excluding the oral contraceptive pill or mild steroidal suppressants) and reported an acute or chronic illness at recruitment. As in previous research on this topic [11,19], volunteers were also required to smoke less than five cigarettes in the daytime and consume less than twenty units of alcohol during the week. In addition, participants were not allowed to have been involved in another study or drug investigation within the previous four weeks. 

### 2.3. Informed Consent

All participants were provided with specific information about the study and required to sign a consent form explaining that they were free to withdraw at any time and confirmed the confidentiality of all information.

### 2.4. Payment

Participants were paid £25 on completion of the study.

### 2.5. Schedule of Testing

All participants were familiarised with the testing procedures and practiced at the tasks. Participants then undertook a baseline session. A pre-requisite for commencement at this stage required all participants to be healthy for a minimum period of one week, prior to the baseline session, at the baseline session, and for the returning test session as a healthy control. On the evenings prior to both the baseline and testing sessions participants were required to limit their alcohol consumption to a maximum of four units and abstain from alcohol on the test days. Smoking and consumption of caffeinated products were prohibited two hours prior to the test sessions. Participation in vigorous exercise was also prohibited on the test days.

All participants were instructed to contact the centre if they began to experience a minor illness e.g., cold, cough, sore throat, or flu. Participants with a cold were not taking any medication (prescribed or over the counter) at the time of repeat testing. If the participants did not experience a minor illness, they were recalled as healthy controls. This was done after a period of ten weeks. 

### 2.6. Symptom Checklist

Volunteers completed a symptom checklist both pre and post performance which assessed the presence and severity of common upper respiratory symptoms (e.g., sore throat, blocked nose, headache, cough, *etc.*). These were rated on a five-point scale from 0 = not present to 4 = very severe. On each test day participants also completed a sleeping and eating log to record sleep duration and quality, food consumption and intake of alcoholic drinks.

### 2.7. Measurement of Nasal Secretion and Sub-lingual Temperature

For the nasal secretion collection, participants were required to clear their nose prior to the performance tests and this tissue was discarded. Participants were provided with a sealed plastic bag containing ten tissues that was pre-weighed. If at any time throughout the 45 minutes of performance tests the participant needed to blow their nose they were instructed to use the tissues provided and place them back into the bag after use. At the end of the test session the bag containing the tissues was reweighed and the weight of the nasal secretions was calculated by subtracting the pre performance weight from the post performance weight. Sub-lingual temperature was also measured.

### 2.8. Mood and Performance Tasks

#### 2.8.1. Mood Ratings

Mood was assessed both pre and post performance using 18 computerised visual analogue mood rating scales which have been widely used and have been the main tool in research on the common cold [33]. Each of the 18 bipolar scales consisted of a pair of adjectives (e.g., drowsy—alert or happy—sad). Participants were instructed to move the cursor from a central position along the horizontal rule, towards either extreme of the scale, until the cursor was at a position representative of their mood state at that exact time. These 18 scales were presented successively. Three main factors were derived from these scales: alertness, hedonic tone and calm. 

#### 2.8.2. Categoric Search Choice Reaction Time Task

In this task the letter A or B was presented but the participant did not known in which of two possible locations the target letter would be displayed. Each trial started with the appearance of two crosses either in the central positions occupied by the non-targets in the focused attention task. The target letter then appeared in place of one of these crosses. On half the trials the target letter A or B was presented alone and on the other half it was accompanied by a distracter, in this task a digit (1–7). Again the number of near/far stimuli, A versus B responses and digit/blank conditions were controlled. Half of the trials led to compatible responses (*i.e.*, the letter A on the left side of the screen, or letter B on the right) whereas the others were incompatible. The nature of the preceding trial was also controlled. This task lasted approximately 8 minutes.

Several variables were measured. The global measures were mean reaction time and accuracy of response when the target was presented alone in either near/far locations. A more specific aspect of choice response was measured, recording choice reaction time and accuracy with which new information was encoded. In addition specific aspects of selective attention were measured. For each of these variables outlined below, mean reaction time and accuracy were calculated. A measure of response organisation (stimulus-response compatibility) was recorded. This refers to the effect of compatibility of the target position and the response key upon reaction time and accuracy. A further measure of place repetition was taken which refers to the effect of target location (*i.e.*, the target appearing in the same or a different place on successive trials). A measure of spatial uncertainty was also taken which describes the extent to which not knowing the location of the target (in near or far locations) hinders both reaction time and accuracy.

#### 2.8.3. Categoric Search Test 2 (with a Variable Delay Between Warning Signals and Stimuli)

This test was similar to the categoric search task described above but included a variable delay after the warning crosses disappeared and the target letter appeared on the screen. Manipulating the task in this way permitted closer examination of organising an appropriate response to the target (motor preparation).

#### 2.8.4. Categoric Search Test 3 (Masked Target)

This test was similar to the categoric search task described above but the target letter was ‘masked’. The stimulus quality was degraded to examine the encoding stage.

In all of the above tasks stimulus-response compatibility was examined.

#### 2.8.5. Serial Recall Task

This task was designed to investigate the articulatory loop of Baddeley’s working memory model. Eight single digits were consecutively presented on the screen at a rate of one per second. Volunteers were required to observe the eight numbers and then write them down in the order in which they had been presented. If they were unsure of a number they were encouraged to guess. This process was repeated five times and written responses recorded on a sheet.

#### 2.8.6. Spatial Memory Task

This task was designed to investigate the visuo-spatial scratch pad described in Baddeley’s working memory model. Volunteers were asked to concentrate on five red buttons displayed on a response box. The red buttons lit up in a randomised sequence consisting of eight lights. The participants were required to observe the sequence and memorise the order in which the buttons lit up. Having observed the light sequence they had to reproduce the sequence by pressing the red buttons in the same order as previously presented. They repeated this process five times.

#### 2.8.7. Running Memory Task

This task was similar to the serial recall task but was designed to examine the central executive of Baddeley’s working memory model. Sequences of consecutive digits were presented to the volunteer at a rate of one per second, however, the length of the digit sequence was unknown and they did not know when the sequence would end. They were required to write down the last six digits of the sequence in the order in which they were presented. Again, if they were unsure of a number they were encouraged to guess. This process was repeated three times and responses were recorded on a sheet. 

## 3. Results

### 3.1. Differences at Baseline

Preliminary analyses using t tests and chi square revealed there were no differences between those participants who developed a minor illness (N = 47) and the healthy controls (N = 54) on measures of demographics, personality and psychosocial factors (stress and social support). Analyses using t tests and chi square indicated no significant differences in demographics, eating, drinking and smoking behaviors. No significant differences were found in personality, psychosocial factors or health related behaviors. Analyses using t tests revealed no differences between those participants who developed a cold and those who remained healthy in terms of symptoms at baseline. No significant differences were found between the ill and healthy groups in terms of sub-lingual temperature at the beginning and end of the baseline session.

### 3.2. Differences at in Symptoms and Signs at Test Session

Analyses using t tests revealed significant differences between the ill and healthy participants on all of the symptoms on the symptoms checklist at the test session. A significant difference was also found between the two groups on the total symptoms score. The table below shows the differences between the two groups.

**Table 1:** Comparisons between symptoms ratings for those with minor illnesses and healthy controls—Visit 2 (Test).

Symptoms	Colds		Healthy		t	p
	Mean (s.d.)	N	Mean (s.d.)	N		
**Total URTI score **	12.06 (5.67)	47	0.91 (1.09)	54	14.17	<0.001
**Pain in chest**	0.19 (0.58)	47	0 (0)	54	2.444	0.016
**Sore throat**	1.49 (1.23)	47	0.04 (0.19)	54	8.003	<0.001
**Headache**	1.28 (1.30)	47	0.09 (0.35)	54	6.067	<0.001
**Sneezing**	1.19 (1.15)	47	0.06 (0.23)	54	6.633	<0.001
**Runny nose**	1.74 (1.28)	47	0.37 (0.56)	54	6.833	<0.001
**Blocked nose**	1.53 (1.23)	47	0.07 (0.33)	54	7.879	<0.001
**Hoarseness**	0.51 (0.75)	47	0.02 (0.14)	54	4.446	<0.001
**Cough**	1.34 (1.18)	47	0.07 (0.33)	54	7.536	<0.001
**Feeling hot/cold**	0.98 (1.15)	47	0.09 (0.29)	54	5.460	<0.001
**Sweating**	0.34 (0.60)	47	0.06 (0.23)	54	3.227	0.002
**Shivering**	0.32 (0.73)	47	0 (0)	54	3.235	0.002
**Fever**	0.26 (0.53)	47	0 (0)	54	3.541	0.001
**Phlegm**	0.89 (0.98)	47	0.04 (0.19)	54	6.272	<0.001

(Symptom scores are the mean ratings, standard deviations in parentheses, from the rating scales 0 = not present to 4 = very severe; Adjusting for the number of analyses carried out means that a p value of < 0.004 should be considered significant).

No significant differences were found between the healthy and ill groups in terms of sub-lingual temperature (Healthy group: mean = 36.3 °C, s.d. = 0.4 °C; Colds group: mean = 36.3 °C, s.d. = 0.4 °C) at the test session. Analyses using t tests revealed significant differences between the healthy and ill group on nasal secretions at the end of the test session (t = 10.58, df = 46, p < 0.005).

In summary, the minor illness group essentially consisted of colds that were confirmed by the symptoms checklist, increased nasal secretion but absence of increased temperature.

### 3.3. Differences between the Healthy and Cold Groups on Measures of Mood and Performance

Analyses of covariance, with the baseline measures as covariates, revealed differences between the healthy and ill participants on pre-performance measures of alertness, hedonic tone and calm. Significant differences between the two groups were also found on post performance measures of alertness and hedonic tone.

**Table 2:** Effects of illness on mood.

Mood	Healthy Mean (S.E)	Cold Mean (S.E)	F & p value
**Pre performance Mood**			
**Alertness**	243.9	168.2	F = 38.54
	(7.2)	(7.9)	p < 0.0001
**Hedonic Tone**	193.13	161.19	F = 19.21
	(4.9)	(5.4)	p < 0.0001
**Calm**	95.47	86.08	F = 7.03
	(2.4)	(2.6)	p < 0.009
**Post performance Mood**			
**Alertness**	215.8	163.2	F = 19.25
	(8.0)	(8.8)	p < 0.0001
**Hedonic Tone**	178.5	148.9	F = 10.30
	(6.2)	(6.8)	p < 0.0018
**Calm**	90.2	85.3	F = 2.46
	(2.1)	(2.3)	p < 0.12

(Scores are the adjusted means from the covariance analyses, s.e. shown in parentheses. High scores = more positive mood. Adjusting for the number of analyses carried out means that a p value of <0.008 should be considered significant).

### 3.4. Effects of Colds on Performance Tests

#### 3.4.1. Effects of Colds on Categoric Search Tasks

Analyses of covariance revealed that those with colds were less accurate across all versions of the categoric search tasks. This reduced accuracy was associated with faster reaction times. Speed of encoding was not altered in those with colds and S-R compatibility did not modify the effects of the illness. The following table shows the separate analyses for the various categoric search tasks. 

#### 3.4.2. Effects of Colds on Working Memory

Analyses of covariance revealed no significant effect of having a cold on working memory, thus confirming the findings of earlier research in this area. Further analyses revealed no significant interaction between healthy and ill groups and serial position (see Table 4).

**Table 3:** Categoric search tasks: speed and accuracy (adjusted means, s.e.s shown in parentheses). (**a**) Comparisons between healthy and ill participants on the basic categoric search task; (**b**) Comparisons between healthy and ill participants on the masked search task; (**c**) Comparisons between healthy and ill participants on the variable fore-period categoric search task.

Performance measure		Healthy Adjusted	Colds Adjusted	F & p value
		mean (s.e.)	mean (s.e)	
**(a)**	**Mean Reaction Time (msec)**	488 (26)	439 (31)	F < 1
	**Number of errors**	6.8 (2.04)	14.0 (2.4)	F = 5.09
				p = 0.02
**(b)**	**Mean Reaction Time (msec)**	555 (8)	549 (9)	F < 1
	**Number of errors**	6.33 (2.04)	14.22 (2.32)	p = 0.01
**(c)**	**Mean Reaction Time (msec)**	533 (24)	487 (27)	F < 1
	**Number of errors**	7.3 (3.48)	16.25 (3.99)	F = 2.85
				p = 0.09

**Table 4:** (**a**) Comparison of healthy and ill participants on serial recall task (proportion correct); (**b**) Comparison of healthy and ill participants on spatial memory task (proportion correct); (**c**) Comparison of healthy and ill participants on running memory task (proportion correct).

Serial Position	(a)		(b)		(c)	
	Healthy	Colds	Healthy	Colds	Healthy	Colds
	Mean	Mean	Mean	Mean	Mean	Mean
**1**	0.88	0.89	0.79	0.79	0.36	0.43
**2**	0.89	0.86	0.78	0.70	0.55	0.53
**3**	0.85	0.84	0.75	0.67	0.69	0.70
**4**	0.87	0.83	0.66	0.67	0.76	0.68
**5**	0.87	0.82	0.67	0.64	0.82	0.76
**6**	0.83	0.83	0.54	0.52	0.82	0.82
**7**	0.79	0.77				
**8**	0.80	0.82				

Further analyses of those with colds examined correlations between symptoms, changes in mood and changes in performance. Symptom severity was correlated with the extent of the negative mood change. However, there were no significant correlations between the performances changes and either the symptom severity or mood changes.

## 4. Overall Discussion

The present study aimed at identifying which cognitive processes might be impaired by upper respiratory tract illnesses. It failed to demonstrate selective effects of minor illnesses on tasks assessing different aspects of working memory and choice reaction time tasks manipulating encoding, central processing and response preparation. However, effects of illness on the speed-error trade-off function were observed, with those with illness showing faster but less accurate performance. Previous research has generally shown slower performance by those with colds and the present results may reflect the greater task difficulty in this study. Further research using techniques examining the speed-error trade-off is required to resolve this issue. Rabbitt [34] discusses tracking models of choice reaction time and argues that a wider range of parameters need to be measured to identify which mechanisms are impaired. Indeed, it may be the case that the volunteers who were ill lost control over the timing of responses and responded too quickly and made a lot of fast errors. Alternatively, if the speed error trade off function is displaced towards slower rts when a person is ill, then they will make more errors if they respond at the normal rate. These alternative views suggest that future studies should not only measure mean correct rts but also mean error rt, the speed error trade off function and the entire distribution of correct and incorrect rts.

Previous studies have largely used simple tasks where the emphasis has been on speed. It is easy to see how the precise composition of the test battery may influence the choice of speed or accuracy. Indeed, while research suggests that having a cold does not impair memory accuracy, there is evidence that it can impair memory speed [35]. It has already been suggested that having a cold leads to many effects which resemble fatigue induced in other ways. Welford [36] suggested that the effects of fatigue are also context dependent. On tasks that involve independent trials fatigue may affect only one stage in the input-output chain, so that one observes a simple decrement in either speed or accuracy. In contrast, serial tasks are monitored less efficiently when a person is fatigued and this may lead to an increase in errors. Indeed, it may be useful to adopt some of the research strategies used to study fatigue to increase our understanding of the effects of minor illnesses on cognition. In addition, it essential to determine how infection influences brain chemistry and to identify the biological mechanisms underlying effects such as those obtained here. It is also important to apply the present approach to study more severe illnesses and to address the implications for real-life activities.
